# Asymmetrical subcortical plasticity entails cognitive progression in older individuals

**DOI:** 10.1111/acel.12857

**Published:** 2018-12-21

**Authors:** Madalena Esteves, Pedro S. Moreira, Paulo Marques, Teresa C. Castanho, Ricardo Magalhães, Liliana Amorim, Carlos Portugal‐Nunes, José M. Soares, Ana Coelho, Armando Almeida, Nadine C. Santos, Nuno Sousa, Hugo Leite‐Almeida

**Affiliations:** ^1^ Life and Health Sciences Research Institute (ICVS), School of Medicine University of Minho Braga Portugal; ^2^ ICVS/3B’s ‐ PT Government Associate Laboratory Braga/Guimarães Portugal; ^3^ Clinical Academic Center – Braga Braga Portugal

**Keywords:** aging, cognition, MMSE, MRI, Stroop, structural laterality

## Abstract

Structural brain asymmetries have been associated with cognition. However, it is not known to what extent neuropsychological parameters and structural laterality covary with aging. Seventy‐five subjects drawn from a larger normal aging cohort were evaluated in terms of MRI and neuropsychological parameters at two moments (M1 and M2), 18 months apart. In this time frame, asymmetry as measured by structural laterality index (ΔLI) was stable regarding both direction and magnitude in all areas. However, a significantly higher dispersion for this variation was observed in subcortical over cortical areas. Subjects with extreme increase in rightward lateralization of the caudate revealed increased M1 to M2 Stroop interference scores, but also a worsening of general cognition (MMSE). In contrast, subjects showing extreme increase in leftward lateralization of the thalamus presented higher increase in Stroop interference scores. In conclusion, while a decline in cognitive function was observed in the entire sample, regional brain asymmetries were relatively stable. Neuropsychological trajectories were associated with laterality changes in subcortical regions.

**Abbreviations**
B: backward
CLTR: consistent long term retrieval
cog: neuropsychological score
D: direct
DR: delayed recall
DS: Digits Span Test
FoV: field of view
GDS: Geriatric Depression Scale
GM: gray matter
L: left
LI: laterality index
LTS: long‐term storage
M: moment
MMSE: Mini‐Mental State Examination
MPRAGE: magnetization‐prepared rapid gradient echo
MRI: Magnetic Resonance Imaging
R: right
SRT: Selective Reminding Test
TE: echo time
WM: white matter
ΔLI: laterality index variation
Δvol: volume variation

## 1 INTRODUCTION

Structural laterality in the human brain has been vastly described (Esteves et al., 2017; Guadalupe et al., 2016; Wyciszkiewicz & Pawlak, 2014; Yamashita et al., 2011), and biological factors such as sex seem to influence these asymmetries (Guadalupe et al., 2016). The planum temporale, for example, shows clear leftward asymmetry (Toga & Thompson, 2003), which seems to be reduced in females (Guadalupe et al., 2015). In aging studies, most research has focused on changes that happen at a functional level where increased activation accompanied by decreased lateralization has systematically been reported. Such alterations have been observed in tasks such as word encoding/retrieval (Cabeza et al., 1997) and working memory (Esteves et al., 2018; Reuter‐Lorenz et al., 2000). Such bilateral activity pattern seems to result from a compensatory recruitment, potentially correlating with good cognitive aging (Cabeza, 2002).

Age‐dependent structural changes have also been described, including a nonlinear alteration of basal ganglia asymmetries (Guadalupe et al., 2016; Wyciszkiewicz & Pawlak, 2014). For example, the putamen, which shows a leftward bias (Esteves et al., 2017; Wyciszkiewicz & Pawlak, 2014), presents decreased asymmetry in males and in younger subjects (Guadalupe et al., 2016), while the globus pallidus suffers a rightward shift with age (Wyciszkiewicz & Pawlak, 2014). The importance of these structural asymmetries arise from associations with neurodegenerative processes like Alzheimer's (Long, Zhang, Liao, Jiang, & Qiu, 2013) and Parkinson's (Lee et al., 2015) diseases, which typically develop at older ages. In fact, structural biases have been correlated with cognitive outcomes such as memory (Esteves et al., 2017; Plessen, Hugdahl, Bansal, Hao, & Peterson, 2014), vocabulary (Esteves et al., 2017; Plessen et al., 2014), and cognitive flexibility (Esteves et al., 2017).

Nonetheless, so far evidence of cognition‐laterality association has been mostly driven from correlational analysis, and causality inferences have been difficult to obtain. One way to surpass this limitation is the utilization of longitudinal approaches, in which a more causal link may be established. Additionally, considering the effects of age on laterality and cognition, specific ranges of ages have to be considered. We have thus explored for the first time the longitudinal association between structural laterality and cognitive traits in an older population. Summarily, neuroimaging and cognitive data were acquired at two time points, 18 months apart. It was hypothesized that variations in cognition would be associated with area‐specific alterations in structural laterality.

## 2 RESULTS

### 2.1 Neuropsychological alterations

Moment (M)1 and 2 cognitive data, as well as comparative statistics is shown in Table 1. From M1 to M2, a statistically significant decline in Selective Reminding Test (SRT), both in the long‐term storage (SRT‐LTS) and delayed recall (SRT‐DR) components, Mini‐Mental State Examination (MMSE) and in the Digits Span Test (DS) direct (DS‐D) and backward (DS‐B) components were found, while no changes were identified in the consistent long‐term retrieval component of the SRT (SRT‐CLTR), either Stroop interference score (Golden/Chafetz) or Geriatric Depression Scale (GDS).

**Table 1:** Neuropsychological characterization of the sample at both moments of evaluation (M1 and M2) and statistical differences between them

	M1	M2	*Z*	Cohen's d	*p*‐value
SRT					
LTS***	28.568 ± 12.659	23.770 ± 14.027	3.493	0.359	< 0.001
CLTR	17.324 ± 12.550	16.743 ± 13.278	0.530	0.045	0.596
DR***	6.000 ± 2.844	4.371 ± 3.108	4.342	0.547	< 0.001
Stroop					
Golden	2.050 ± 7.553	3.082 ± 8.174	−0.802	0.131	0.422
Chafetz	−6.548 ± 8.835	−5.288 ± 8.422	−0.665	0.146	0.506
MMSE***	27.085 ± 3.193	25.972 ± 3.216	3.942	0.347	< 0.001
GDS	11.448 ± 6.898	10.241 ± 6.878	1.737	0.175	0.082
DS					
D***	7.865 ± 2.259	7.041 ± 1.926	3.953	0.393	< 0.001
B*	4.662 ± 2.512	4.257 ± 2.000	2.007	0.179	0.045

Notes

Data are shown as mean ± standard deviation. Asterisks represent statistically significant differences between M1 and M2.

B: backward; CLTR: consistent long term retrieval; D: direct; DR: delayed recall; DS: Digits Span Test; GDS: Geriatric Depression Scale; LTS: long term storage; M1: moment 1; M2: moment 2; MMSE: Mini‐Mental State Examination; SRT: Selective Reminding Test.

* *p* < 0.05;

*** *p* < 0.001.

### 2.2 Changes in laterality

**Figure 1:**
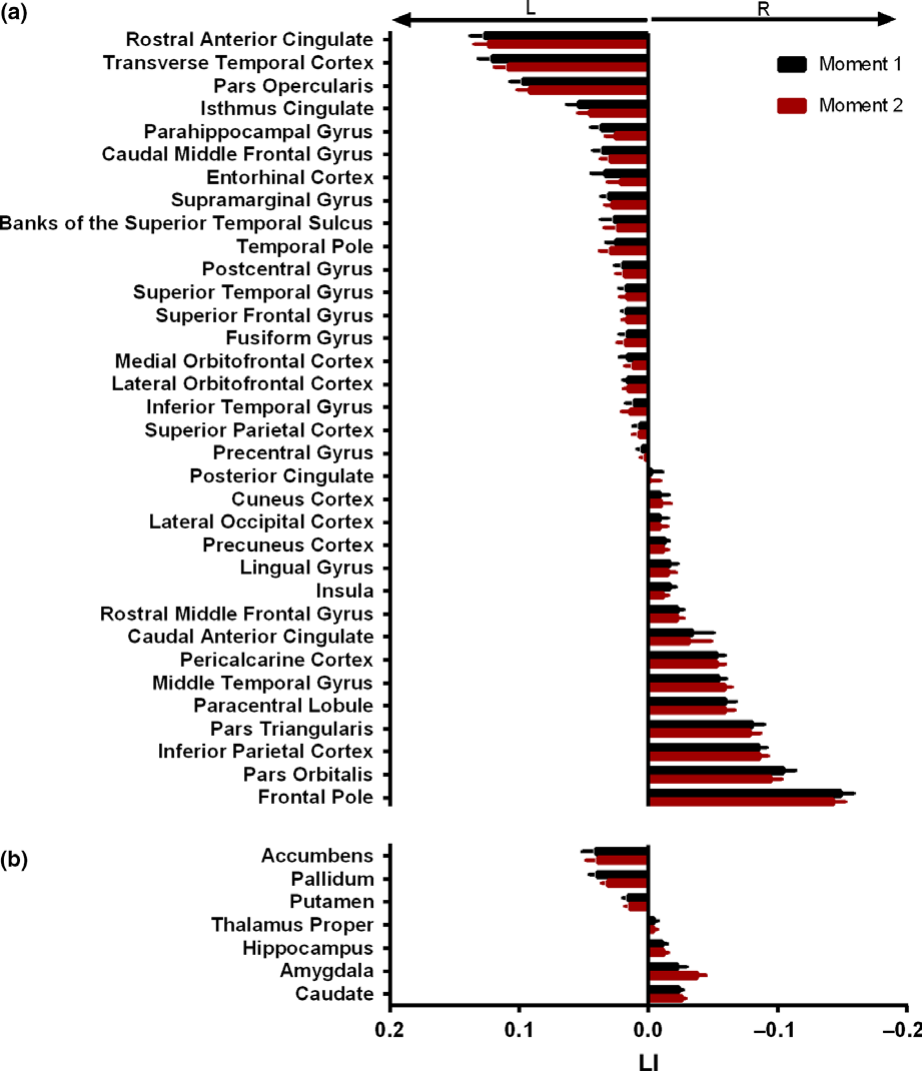
Average structural laterality at M1 and M2. Structural laterality of cortical gray matter (a) and subcortical (b) areas at M1 and M2. Bar graphs show mean and standard error of the mean (*SEM*) and are organized from highest to lowest LI at M1. Positive and negative values represent left and rightward laterality, respectively, and are represented on the left and right side of the graphs. L, left; R, right; LI, laterality index; M1, moment 1; M2, moment 2

Analysis of the laterality index (LI) at both M1 and M2 revealed ubiquitous asymmetries in both directions (Figure 1a,b, Supporting information Table S1) with no differences in average laterality between the two moments (Supporting information Table S1). In fact, among 41 areas, only six were found to be lateralized at M1 but not at M2, namely the insula, parahippocampal, postcentral, and lingual gyri, while temporal pole and hippocampus were found to be lateralized at M2 but not at M1 (Supporting information Table S1).

**Figure 2:**
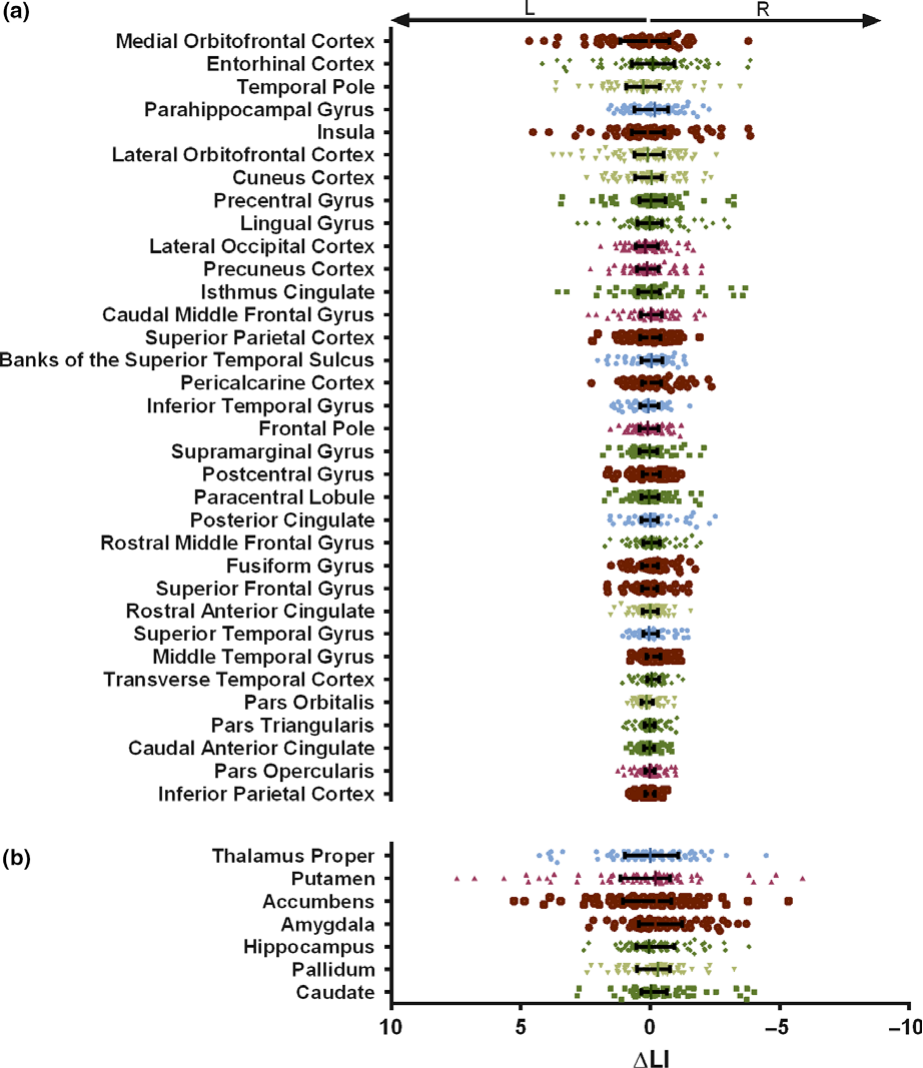
Individual values of structural laterality variation. Individual values of ΔLI for cortical gray matter (a) and subcortical (b) areas. Dots represent individual values, and lines represent mean and interquartile range. Areas are organized from highest to lowest dispersion. Positive and negative values represent left and rightward evolution, respectively, and are represented on the left and right side of the graphs. L, left; R, right; ΔLI, variation of laterality index

Average ΔLI was thus approximately 0 in all areas (Figure 2a,b) and was not influenced by cognitive performance group (i.e. good or poor cognitive performers), age, or sex. Nonetheless, dispersion of values was area‐dependent, and interquartile range was higher in subcortical rather than cortical GM areas (Figure 2b vs. a ‐ *Z* = 3.586; Cohen's *d* = 2.185; *p* < 0.001). Further analysis was therefore focused in subcortical regions.

### 2.3 Left/Right volumes equally contribute to variation of subcortical laterality

**Figure 3:**
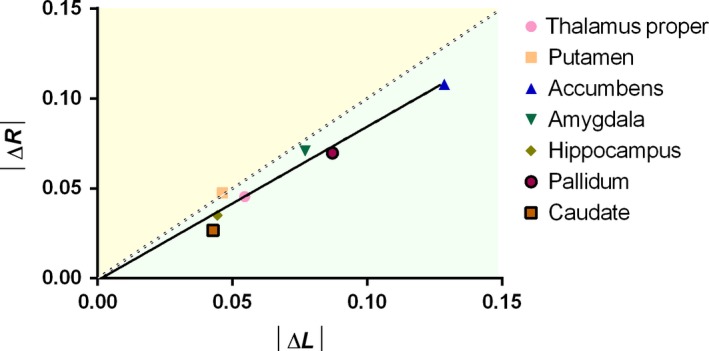
Graphical representation of left and right variation influence for ΔLI. Representative graph of similar left and right subcortical volume variation in the right and left categories**. **Individual dots represent average absolute variation of left and right area volume in the extreme (right and left) categories. Full line represents the linear regression for these values, and dotted line represents perfect │ΔL│ − │ΔR│ correlation (slope = 1). Green and yellow areas represent, respectively, areas of higher │ΔL│ or │ΔR│. │ΔL│ = absolute value of M1 to M2 left area volume variation, │ΔR│ = absolute value of M1 to M2 right area volume variation

The contribution of left and right volumes variation to ΔLI in individuals whose LI evolved to the left, to the right, or maintained unaltered (left, right, and nil categories, respectively) was evaluated using logistic regression. In all areas, variation of left and right volumes significantly contributed to this categorization in the same order of magnitude but in inverse direction, that is, an increase in right area volume increased the probability of being placed in the right category and vice versa for increase in left area volume (Table 2). This is graphically represented in Figure 3, which shows the similar average left and right volume variations in the extreme (left and right) categories (ΔR = 0.8546*ΔL − 0.001; *R* ^2^ = 0.972; *p* < 0.001).

**Table 2:** Left and right subcortical volume variation influence in the establishment of left, right and nil categories

	OR	95% CI		*p*‐value
		Lower	Upper	
Thalamus proper				
ΔR	1.581E−56	2.772E−83	9.022E−30	<0.001
ΔL	2.362E+55	1.454E+29	3.836E+81	<0.001
Putamen				
ΔR	3.228E−33	2.344E−48	4.445E−18	<0.001
ΔL	2.734E+45	1.478E+24	5.059E+66	<0.001
Accumbens				
ΔR	9.720E−21	4.795E−31	1.970E−10	<0.001
ΔL	7.878E+25	2.155E+12	2.880E+39	<0.001
Amygdala				
ΔR	1.352E−37	4.367E−60	4.183E−15	0.001
ΔL	1.630E+40	9.522E+16	2.792E+63	0.001
Hippocampus				
ΔR	2.858E−73	2.623E−111	3.113E−35	<0.001
ΔL	2.648E+75	5.559E+34	1.261E+116	<0.001
Pallidum				
ΔR	2.994E−18	9.164E−27	9.785E−10	<0.001
ΔL	2.435E+20	4.299E+10	1.380E+30	<0.001
Caudate				
ΔR	3.078E−97	1.400E−130	6.769E−64	<0.001
ΔL	2.229E+98	1.618E+65	3.071E+131	<0.001

Notes

CI: confidence interval; OR: odds ratio; ΔL: variation of left volume (M1 to M2);ΔR: variation of right volume (M1 to M2).

### 2.4 Neuropsychological changes associate with subcortical variation of laterality

**Figure 4:**
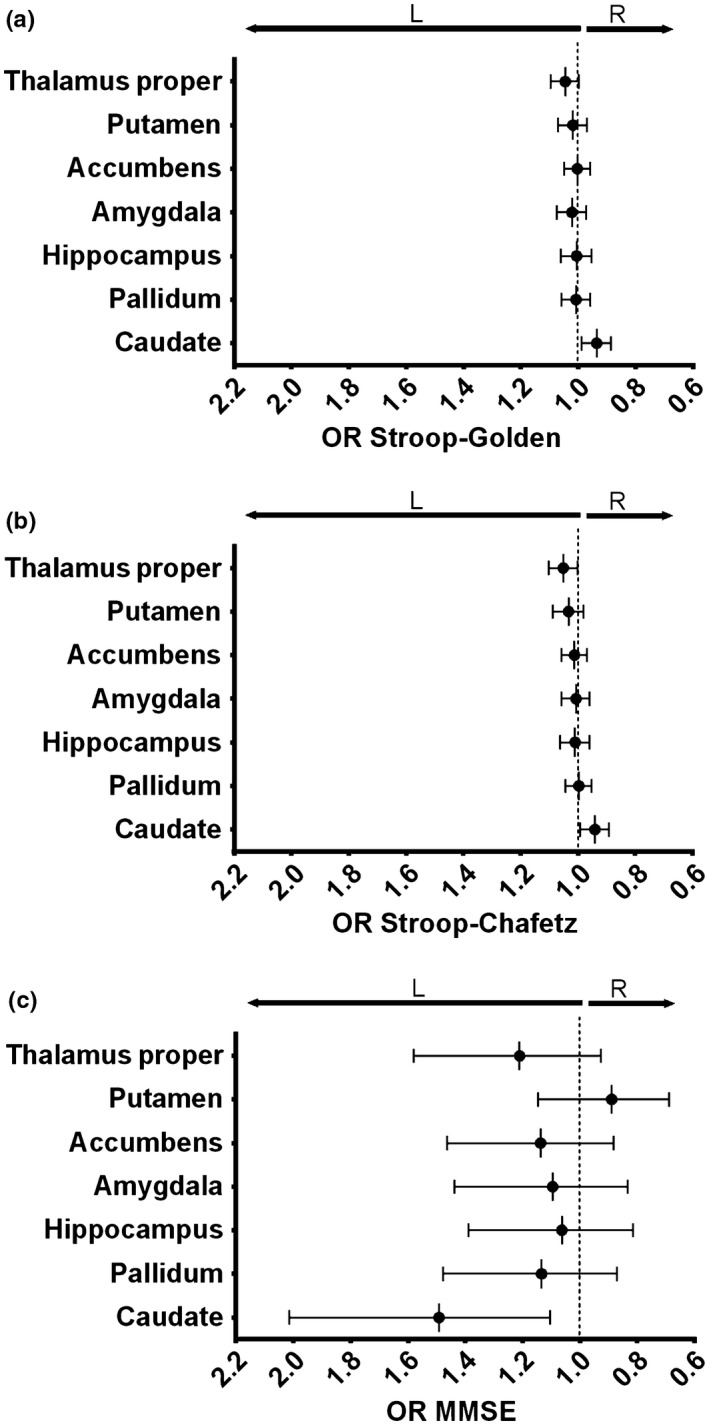
Graphical representation of the neuropsychological M1 to M2 variation influence in subcortical ΔLI. The graphs depict OR and 95% CI of (a) Stroop's Golden Index, (b) Stroop's Chafetz Index, and (c) MMSE M1 to M2 variation's influence on ΔLI categorization for each subcortical area. OR higher and lower than 1 represent leftward and rightward evolution of ΔLI and are, respectively, represented on the left and right side of the graphs. Increased Stroop (Golden or Chafetz indices) and MMSE scores means lower Stroop interference effect and higher general cognition, respectively. Regressions are controlled for total gray matter change as a proxy for aging. L, left; R, right; OR, odds ratio; MMSE, Mini‐Mental State Examination; CI, confidence interval

The association between M1 to M2 neuropsychological variation and ΔLI was assessed. As stated above, on average M1 to M2 LI was stable and therefore extreme variants on each direction (left and right) and non‐variants (nil) were analyzed in a logistic regression approach. When controlling only for GM change as a proxy for aging, better M1 to M2 performance in the Stroop test (increased Chafetz interference score) was associated with leftward variation of thalamus' volume. Leftward variation of the caudate was associated with worse (lower) Stroop interference scores and better (higher) general cognition in the MMSE. Data can be seen in Supporting information Table S2 and Figure 4: (a) an increase of 1 point on Stroop's Golden index was associated with a 6% increase in the probability of caudate's LI varying to the right (negative ΔLI) (Figure 4a ‐ OR = 0.935; CI = 0.886 to 0.988; *p* = 0.016); (b) a similar association was found with the Stroop's Chafetz index (Figure 4b ‐ OR = 0.940; CI = 0.891–0.992; *p* = 0.025) while the same index variation was associated with a 5% increase in the probability of thalamus' LI varying to the left (positive ΔLI) (Figure 4b ‐ OR = 1.051; CI = 1.002–1.102; *p* = 0.040); and (c) a point increase in the MMSE score was associated with a striking 49% probability of left (positive) variation in the caudate LI (Figure 4c ‐ OR = 1.491; CI = 1.105–2.014; *p* = 0.009). Importantly, all results were maintained when controlling the analyses for age, sex and cognitive performance group (good and poor performers; Supporting information Table S2). No associations were found with SRT, GDS or DS (Supporting information Table S2).

### 2.5 Neuropsychological changes do not associate with subcortical left/right volume variations

**Figure 5:**
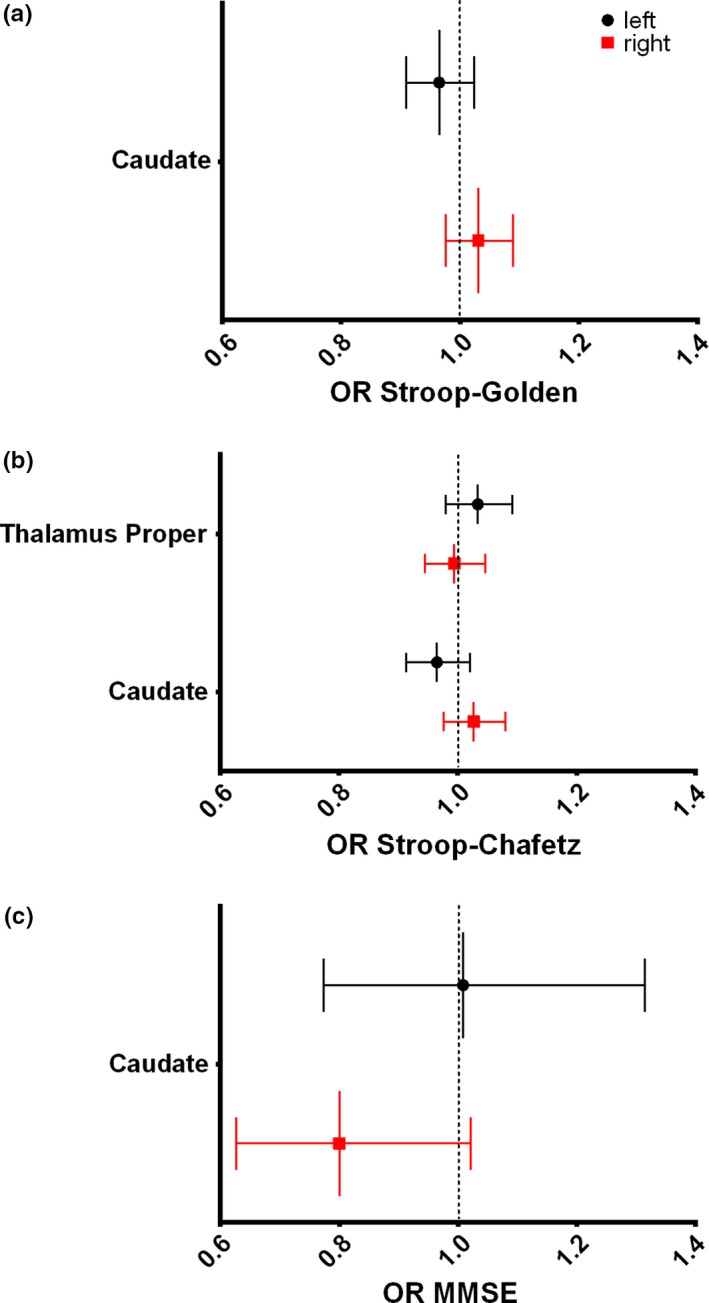
Graphical representation of the neuropsychological M1 to M2 variation influence in subcortical left and right volume changes. The graphs depict OR and 95% CI of (a) Stroop's Golden Index, (b) Stroop's Chafetz Index, and (c) MMSE M1 to M2 variation's influence on volume categorization for each subcortical area, that is, decrease, maintenance, or increase in volume. Increased Stroop (Golden or Chafetz indices) and MMSE scores means lower Stroop interference effect and higher general cognition, respectively. Associations with left and right volume variations are depicted in black and red, respectively. Regressions are controlled for total gray matter change as a proxy for aging. OR, odds ratio; MMSE, Mini‐Mental State Examination; CI, confidence interval

Associations between neuropsychological changes and individual variation of left and right volumes were verified for regions in which correlations with laterality were found in the above section. This aimed to determine whether these results could in fact be attributed to laterality rather than individual volumes. Because M1 to M2 volume variation did not differ from 0 (thalamus left *p* = 0.237; thalamus right *p* = 0.099; caudate left *p* = 0.132; caudate right *p* = 0.378), a similar percentile strategy was applied: reduction, maintenance, or increase in volume were predicted in a logistic regression analysis (Figure 5).

In all analyses, none of the associations with individual left or right volumes achieved statistical significance: Stroop's Golden Index and caudate – OR = 0.965, CI = 0.909–1.024, *p* = 0.243 for left volume and OR = 1.031, CI = 0.977–1.089, *p* = 0.269 for right (Figure 5a); Stroop's Chafetz Index and thalamus – OR = 1.034, CI = 0.979–1.092, *p* = 0.229 for left volume and OR = 0.994, CI = 0.944–1.047, *p* = 0.830 for right (Figure 5b); Stroop's Chafetz Index and caudate – OR = 0.965, CI = 0.913–1.020, *p* = 0.210 for left volume and OR = 1.027, CI = 0.976–1.080, *p* = 0.307 for right (Figure 5b); and MMSE and caudate – OR = 1.009, CI = 0.774–1.314, *p* = 0.949 for left volume and OR = 0.800, CI = 0.627–1.022, *p* = 0.074 for right (Figure 5c). Of note, in all cases, the trend followed the results found in the laterality results, that is, whenever increase in neuropsychological score was associated with rightward laterality variation, a trend toward right increase and left decrease was found (and vice versa for associations with leftward variation).

## 3 DISCUSSION

Aiming to study asymmetrical plasticity and respective neuropsychological correlates, herein, we evaluated 75 older individuals in two different moments. Data analysis indicates that, in older individuals, an 18‐month time window was sufficient to observe a general cognitive decline, but no average changes in structural laterality. In subcortical areas, individuals were more heterogeneous regarding LI variation between the two moments. Interestingly, counterpart areas in the left and right hemisphere contributed nearly equally for this variation (varying in opposing directions) suggesting some degree of organization in the phenomena and excluding potential local neuropathological events. Importantly, in the caudate and thalamus laterality, variations (M1 to M2) were associated with the course of mental flexibility and general cognition, which could not be attributed to individual left and right volume variation.

With aging, there is a general atrophy of GM (Hedden & Gabrieli, 2004). The scale of these reductions is area‐dependent; for instance, *per* decade, lateral pre‐frontal cortex reduces its volume in 5% (Raz, Gunning‐Dixon, et al., 2004); the hippocampus may reach a 6% reduction at higher ages (Raz, Rodrigue, Head, Kennedy, & Acker, 2004). These reductions may translate into age‐dependent changes in laterality but results in this domain have been inconsistent. Both asymmetry reductions (Long et al., 2013) and increases—caudate (Yamashita et al., 2011) and cortical thickness (Plessen et al., 2014)—have been reported, while other authors have found no effects of age on brain asymmetries (Raz, Gunning‐Dixon, et al., 2004; Raz, Rodrigue, et al., 2004). Two important factors contributing to these disparities may be the strategy used to assess laterality (Taki et al., 2011) and on the range of ages evaluated (i.e. it may not be a linear change (Guadalupe et al., 2016; Zhou, Lebel, Evans, & Beaulieu, 2013)). Considering the small time window between the 2 evaluations of our study, it is not surprising that we were unable to find differences in volumetric laterality. Additionally, reproducing the results obtained in the cross‐sectional analysis of this cohort (M. Esteves et al., 2017), laterality variation was not associated with sex, age, or cognitive performance group (i.e. good or poor cognitive performers).

On the other hand, the striking difference between dispersion of cortical and subcortical laterality indices was not expected. This showed that, although the average was maintained, a higher number of individuals experienced variations in subcortical asymmetries. In fact, some subcortical areas were previously shown to have high rates of atrophy during healthy aging (Fjell et al., 2009). Variations in side‐specificity of this atrophy may be associated with increased dispersion laterality values' trajectory. Of importance, we were able to show that left or right variation of subcortical laterality was not due to local phenomena, but was rather associated with opposite patterns in the two hemispheres (i.e. left and right volume change equally contributed for the LI variation).

It is widely accepted that aging induces a decline of cognitive functions such as the encoding of episodic memories and processing speed, while others like semantic memory and emotional processing remain mostly unaltered (Hedden & Gabrieli, 2004). Accordingly, in the time window of this study, we observed a general decline in MMSE, which was negatively correlated with leftward variation of caudate's LI (i.e. MMSE increase was associated with increased leftward lateralization). This area has been vastly shown to be reduced in diseases associated with cognitive decline such as Parkinson's disease (Apostolova et al., 2012) or Alzheimer's disease (Barber, McKeith, Ballard, & O'Brien, 2002; Looi et al., 2008; Madsen et al., 2010). Additionally, both left and right caudate stroke was shown to induce cognitive decline (Bokura & Robinson, 1997) but side‐specific associations have been found. In fact, in accordance with the overall rightward asymmetry of the caudate in our healthy cohort, right volume (Apostolova et al., 2010) and rightward asymmetry of this area (Madsen et al., 2010) have been previously described as higher in non‐demented rather than demented patients. Also, other authors have described reduced left (but not right) caudate volume in demented patients (Barber et al., 2002) and a positive correlation between left (but not right) caudate volume and MMSE score, when assessing different types of cognitive decline (Looi et al., 2008). It is important to notice that we were, to the best of our knowledge, the first to assess a longitudinal index that measures left and right differences rather than absolute volumes. In fact, in our cohort, caudate's left/right balance, rather than the absolute volumes, better associated with cognitive decline, and we may speculate that it should be relevant for disease onset.

No alterations in either measure of Stroop interference effect (Golden and Chafetz) were observed between M1 and M2. Regarding these tests, the literature presents conflicting evidence of an age effect. Indeed, while most studies show an interference increase with age (Davidson, Zacks, & Williams, 2003; Troyer, Leach, & Strauss, 2006), others (Langenecker, Nielson, & Rao, 2004), including a meta‐analysis (Verhaeghen & De Meersman, 1998), found no evidence of such effect. It is important to stress that these are cross‐sectional studies, using wider age ranges than the 18‐month interval used in our study. We here applied two different Stroop interference calculations: Golden (Lansbergen, Kenemans, & van Engeland, 2007) and Chafetz (Chafetz & Matthews, 2004) indices. These retrieved slightly different results with increased interference score (i.e. decreased interference) in the Chafetz index associated with thalamus and caudate leftward and rightward trajectories, respectively, while only the latter was found in association with the Golden index. Indeed, while these two indices are expected to measure the same effect, there is no consensus in the definition of a gold standard, and, as the formula for index calculation is different, small differences in the results were expected. Additionally, it should be noted that, although the association between the Golden index and thalamus trajectory was not significant, the direction of the trend was similar to the Chafetz index. Performance in Stroop test has been classically associated with activation of frontal, cingulate, and temporal areas (Langenecker et al., 2004; Peterson et al., 1999), although relatively consistent findings in caudate and thalamic regions have also been described (Langenecker et al., 2004; Van Der Werf et al., 2001)—see also (Peterson et al., 1999) for comparison of different studies. Additionally, left but not right caudate has been shown to be activated in incongruent vs. congruent Stroop contrast (Langenecker et al., 2004), which may be related to its role in the switch between these two conditions, as the left (but not right) caudate head reduces its BOLD signal during this transition (Ali, Green, Kherif, Devlin, & Price, 2010). On the other hand, Cai et al. (2016) have shown in individuals with internet gaming disorder that increased errors in incongruent Stroop are positively correlated with right caudate volume. Regarding the thalamus, our group has recently observed an association between Stroop words and colors and thalamus laterality (Esteves et al., 2017) in a transversal analysis of this same cohort. Our current results seem to reinforce this previous finding. Altogether, the sparse literature in the matter seems to agree with our data, showing a differential role of left and right caudate and thalamus in the Stroop interference effect.

No other regions showed associations with either cognitive or emotional changes. In the case of the nucleus accumbens or the amygdala, for instance, it might be speculated that this absence may be due to small M1 to M2 changes in neuropsychological scores related with mood. In this case, the time window of our study could be masking a possible association. However, it should be noted that the functions traditionally attributed to these regions are not necessarily asymmetry‐dependent.

In conclusion, brain asymmetries (Plessen et al., 2014; Zhou et al., 2013) and cognitive performance (Hedden & Gabrieli, 2004) change with age, raising the hypothesis that these two phenomena could be associated. However, as these changes do not seem to follow a linear trend (Guadalupe et al., 2016; Zhou et al., 2013), assessment of a stringent age category is necessary, and the characteristic cognitive decline of aged individuals makes them prime candidates for such evaluation. Here, despite the absence of change in average structural laterality in the 18‐month time frame, it is shown that intra‐individual variability in this measure was higher in subcortical rather than cortical areas. Additionally, caudate and thalamus laterality variations were associated with changes in mental flexibility and general cognition.

## 4 EXPERIMENTAL PROCEDURES

### 4.1 Ethics statement

Procedures were performed according to the Declaration of Helsinki and were approved by national and local ethics committees. All volunteers signed informed consent.

### 4.2 Subjects

Subjects were evaluated at two time points 18 months apart (mean ± standard deviation 561 ± 55 days; minimum 502; maximum 791). The sample used in this study was withdrawn from the Switchbox project and selection for the first moment of evaluation (M1) has been previously described (Esteves et al., 2017; Marques, Soares, Magalhaes, Santos, & Sousa, 2016). Briefly, a sample representative of the older Portuguese population was selected from the Guimarães and Vizela health authority registries (*n* = 1,051) (Santos et al., 2013). Primary exclusion criteria (at both time points) included incapacity to understand the informed consent, choice to withdraw from the study and/or diagnosed dementia, neuropsychiatric, or neurodegenerative disorder. Cognitive data were used in order to perform Principal Component Analysis followed by cluster analysis, in which four clusters were identified. A total of 120 subjects belonging to the best and worst cognitive performers, balanced for sex and age, were selected for further characterization at M1, including Magnetic Resonance Imaging (MRI). All subjects were right handed. At the second time point (M2), two individuals could not be contacted, six were unable to attend the assessment, and 26 met exclusion criteria (17 by decision to withdraw from the study). In total, 86 subjects agreed to participate in the study. Nine refused to perform MRI (at either the first or second time points), one had brain lesions detected at MRI M2, and one was excluded due to movement artifacts at M2. The final population for longitudinal assessment thus included 75 individuals, from which 36 were females, 47 belonged to the good performers group, average education was 5.9 ± 4.1 years (mean ± standard deviation; minimum 0; maximum 17), and average age at M1 was 64.6 ± 7.8 years old (mean ± standard deviation; minimum 51; maximum 82). Further characterization of the cohort according to cognitive performance group and sex can be consulted in Supporting information Table S3.

### 4.3 Cognitive assessment

A team of trained psychologists applied and scored all neuropsychological tests as previously described (Santos et al., 2014) at both time points aiming to assess memory, executive function, general cognition, and mood. The memory domain, more specifically verbal learning and memory, was evaluated through the SRT (Buschke, Sliwinski, Kuslansky, & Lipton, 1995). In this test, a list of 12 words is read to the participant, who is asked to repeat as many as possible on a first trial. In the five trials that follow, only the words not recalled on the previous one are read back to the participant. Three different components are evaluated: LTS is considered when a given word is recalled in two consecutive trials; CLTR is considered when words are recalled in all subsequent trials; and DR consists of words recalled after 20 min.

Executive function was assessed through the Stroop test (Strauss, Sherman, & Spreen, 2006) and the Digits Span Test (Wechsler, 1997). The first aimed at assessing selective attention, cognitive flexibility, and response inhibition utilizing two different composites: the Golden (Lansbergen et al., 2007) and Chafetz (Chafetz & Matthews, 2004) indices, which evaluate the level of interference when the name of a color is written in a different color ink (e.g. the word blue written in red ink; higher score means decreased Stroop interference). While both indices are expected to measure the same effect, there is no general consensus in terms of defining one as gold standard, and therefore, we utilized both, aiming to achieve higher internal control. The second executive function test, the Digits Span Test, consists of a progressively longer list of numbers that is read to the participant. The participant is then asked to immediately repeat the list in the same order, assessing attention (DS‐D), or in the reverse order, measuring working memory/executive function (DS‐B).

General cognition was evaluated using the MMSE (Guerreiro et al., 1994), a questionnaire that provides a short assessment of orientation, memory, attention, language, verbal comprehension, writing, and visual construction. A second questionnaire, the GDS, evaluated depressive mood (Yesavage et al., 1982).

### 4.4 Image acquisition and analysis

A clinically approved Siemens MagnetomAvanto 1.5 T (Siemens Medical Solutions, Elangen, Germany) with a 12‐channel receive‐only Siemens head coil was used to perform all acquisitions at Hospital de Braga (Braga, Portugal). A scan using a T1‐weighted magnetization‐prepared rapid gradient echo (MPRAGE) sequence with the following parameters: repetition time (TR) = 2,730 ms, echo time (TE) = 3.5 ms, flip angle = 7°, field of view (FoV) = 256 mm, 176 sagittal slices, isotropic resolution of 1 mm, and no slice‐gap. All raw acquisitions were visually inspected by a certified neuroradiologist, confirming the absence of brain lesions and critical artifacts. Structural data were processed using the semi‐automated workflow implemented in FreeSurfer v5.10 (https://surfer.nmr.mgh.harvard.edu/) which has been thoroughly described and continuously updated (Desikan et al., 2006; Fischl et al., 2002). The 31 processing steps were run, including spatial normalization to Talairach standard space, skull stripping, intensity normalization, tessellation of gray matter (GM)‐white matter (WM) boundary and segmentation of cortical, subcortical, and WM regions. This pipeline has been validated against manual segmentation (Fischl et al., 2002). Only subcortical and cortical gray matter (GM) volumes according to the Desikan atlas were considered (Desikan et al., 2006).

### 4.5 Data analysis

All statistical analyses were performed on Matlab R2009b software (The MathWorks, Inc., Natick, Massachusetts, United States). A threshold of *p* < 0.05 for statistical significance was considered and Bonferroni‐Holm multiple comparison correction was applied when whole brain analyses were performed to control for the family wise error rate. Whenever normality assumptions were not met, nonparametric testing was performed. All graphs were attained using Prism 6 software (GraphPad Software, Inc., La Jolla, USA). For each cortical GM and subcortical area, a LI was calculated as LI = (L‐R)/(L + R), where L corresponds to left hemisphere area volume and R corresponds to right area volume. Positive values indicate L > R and negative values indicate L < R, while the denominator provides normalization for total area volume. Variation of LI (ΔLI) was defined as ΔLI = (LI_M2 − LI_M1)/│LI_M1│, where LI_M2 and LI_M1 correspond to LI on the second and first moment of evaluation, respectively, and │LI_M1│ is the absolute value of LI_M1. Positive values indicate variation to the left (i.e. at M2, the area was more asymmetric to the left, when comparing with M1), and negative values indicate variation to the right. The denominator provides normalization to basal laterality levels. Variation of left and right volumes (Δvol) was defined in a similar fashion: Δvol = (vol_M2 − vol_M1)/vol_M1; variation of neuropsychological scores was defined as cog_M2‐cog_M1, where cog_M2 and cog_M1 correspond, respectively, to score at M2 or M1. Positive and negative values indicate an increase and decrease of neuropsychological score, respectively .

Determination of M1 to M2 variation (cog and LI) was performed using paired nonparametric comparisons, as normality could not be confirmed, and analysis of potential influence of demographic data on ΔLI utilized linear regression models. Inter‐individual dispersion of ΔLI was assessed using the interquartile range. All analyses in which neuropsychological variation was the independent variable of interest were performed using ordinal logistic regression and were always corrected for variation of total gray matter (GM) as a proxy for aging. Categories for analyses in which the dependent variable was ΔLI were also based on percentiles and included the lower (right variation), middle (no variation), and higher (left variation) 25% of ΔLI (right, nil, and left categories, respectively). Left variation was always the reference category. Categories for analyses in which the dependent variable was Δvol included the lower (reduction), middle (maintenance), and higher (increase) 25% of volume variation.

## CONFLICT OF INTEREST

The authors declare no conflict of interest.

## AUTHOR CONTRIBUTIONS

M.E., N.S., and H.L.A involved in conceptualization; M.E., P.S.M., and H.L.A. performed formal analysis; M.E., P.S.M., P.M., T.C.C., R.M., L.A., C.P.N., J.M.S., and H.L.A. involved in investigation; M.E. and H.L.A wrote the original draft; M.E., P.S.M., P.M., T.C.C., R.M., L.A., C.P.N., J.M.S. A.C., A.A., N.C.S., N.S., and H.L.A wrote, reviewed, and edited the manuscript; N.S and H.L.A involved in supervision.
