# Supplementary material for: Asymmetrical subcortical plasticity entails cognitive progression in older individuals
Source: Aging Cell. 2018 Dec 21;18(1):e12857. doi: 10.1111/acel.12857 (PMC6351824; doi:10.1111/acel.12857)
Supplement: Supplementary file 1 [file ACEL-18-e12857-s001.docx]

**Table S1 - Laterality statistics**

| area | | LI vs 0 | | | | | | LI M1 vs M2 | | |
| --- | --- | --- | --- | --- | --- | --- | --- | --- | --- | --- |
|  |  | M1 | | | M2 | | |  |  |  |
|  |  | Z | effect size (r) | corrected p-value | Z | effect size (r) | corrected p-value | Z | effect size (cohen's d) | corrected p-value |
| Cortical GM | Rostral Anterior Cingulate | 7.011 | 0.815 | <0.001 | 7.018 | 0.810 | <0.001 | 0.539 | 0.030 | 10.557 |
|  | Transverse Temporal Cortex | 6.791 | 0.784 | <0.001 | 6.849 | 0.791 | <0.001 | 2.158 | 0.134 | 1.053 |
|  | Pars Opercularis | 6.706 | 0.774 | <0.001 | 6.886 | 0.806 | <0.001 | 1.119 | 0.065 | 6.581 |
|  | Isthmus Cingulate | 5.239 | 0.609 | <0.001 | 4.008 | 0.463 | 0.002 | 1.463 | 0.107 | 4.163 |
|  | Parahippocampal Gyrus | 3.765 | 0.435 | 0.004 | 2.783 | 0.321 | 0.086 | 2.028 | 0.167 | 1.403 |
|  | Caudal Middle Frontal Gyrus | 3.702 | 0.427 | 0.004 | 3.638 | 0.420 | 0.006 | 1.751 | 0.088 | 2.399 |
|  | Entorhinal Cortex | 2.820 | 0.328 | 0.067 | 1.848 | 0.213 | 0.517 | 0.729 | 0.134 | 10.256 |
|  | Supramarginal Gyrus | 4.721 | 0.545 | <0.001 | 4.156 | 0.480 | 0.001 | 0.702 | 0.062 | 10.256 |
|  | Banks of the Superior Temporal Sulcus | 2.350 | 0.271 | 0.207 | 2.044 | 0.236 | 0.410 | 0.644 | 0.030 | 10.394 |
|  | Temporal Pole | 2.804 | 0.324 | 0.067 | 3.464 | 0.400 | 0.010 | 1.109 | 0.062 | 6.581 |
|  | Postcentral Gyrus | 2.962 | 0.342 | 0.046 | 2.387 | 0.276 | 0.204 | 0.388 | 0.020 | 10.322 |
|  | Superior Temporal Gyrus | 3.824 | 0.448 | 0.003 | 3.257 | 0.381 | 0.020 | 0.312 | 0.013 | 9.016 |
|  | Superior Frontal Gyrus | 4.684 | 0.545 | <0.001 | 4.140 | 0.478 | 0.001 | 0.477 | 0.018 | 10.136 |
|  | Fusiform Gyrus | 2.191 | 0.253 | 0.256 | 2.429 | 0.280 | 0.204 | 0.380 | 0.027 | 9.777 |
|  | Medial Orbitofrontal Cortex | 2.302 | 0.266 | 0.213 | 2.443 | 0.284 | 0.204 | 0.544 | 0.079 | 10.557 |
|  | Lateral Orbitofrontal Cortex | 3.770 | 0.435 | 0.004 | 3.844 | 0.444 | 0.003 | 0.256 | 0.015 | 6.301 |
|  | Inferior Temporal Gyrus | 1.288 | 0.149 | 0.790 | 1.901 | 0.220 | 0.516 | 1.177 | 0.053 | 6.218 |
|  | Superior Parietal Cortex | 1.547 | 0.179 | 0.731 | 1.537 | 0.177 | 0.519 | 0.073 | 0.011 | 2.760 |
|  | Precentral Gyrus | 1.447 | 0.167 | 0.740 | 0.840 | 0.097 | 0.745 | 1.212 | 0.079 | 6.088 |
|  | Posterior Cingulate | 0.444 | 0.052 | 0.847 | 0.892 | 0.104 | 0.914 | 0.305 | 0.020 | 8.309 |
|  | Cuneus Cortex | 1.161 | 0.135 | 0.790 | 1.026 | 0.119 | 0.914 | 0.191 | 0.020 | 4.972 |
|  | Lateral Occipital Cortex | 1.845 | 0.214 | 0.475 | 1.770 | 0.206 | 0.537 | 0.053 | 0.007 | 1.884 |
|  | Precuneus Cortex | 3.553 | 0.413 | 0.007 | 3.059 | 0.358 | 0.038 | 1.102 | 0.039 | 6.419 |
|  | Lingual Gyrus | 3.181 | 0.370 | 0.023 | 2.704 | 0.312 | 0.103 | 0.288 | 0.026 | 7.603 |
|  | Insula | 3.406 | 0.393 | 0.012 | 2.329 | 0.269 | 0.219 | 1.299 | 0.144 | 5.430 |
|  | Rostral Middle Frontal Gyrus | 4.156 | 0.480 | 0.001 | 4.145 | 0.479 | 0.001 | 0.317 | 0.006 | 9.149 |
|  | Caudal Anterior Cingulate | 1.885 | 0.218 | 0.475 | 1.626 | 0.188 | 0.540 | 0.401 | 0.015 | 10.322 |
|  | Pericalcarine Cortex | 6.521 | 0.758 | <0.001 | 6.220 | 0.718 | <0.001 | 0.100 | 0.008 | 3.546 |
|  | Middle Temporal Gyrus | 6.419 | 0.746 | <0.001 | 6.759 | 0.780 | <0.001 | 1.965 | 0.099 | 1.580 |
|  | Paracentral Lobule | 5.719 | 0.660 | <0.001 | 5.840 | 0.674 | <0.001 | 0.143 | 0.006 | 4.242 |
|  | Pars Triangularis | 6.426 | 0.742 | <0.001 | 6.585 | 0.760 | <0.001 | 0.217 | 0.024 | 5.586 |
|  | Inferior Parietal Cortex | 7.356 | 0.855 | <0.001 | 7.297 | 0.848 | <0.001 | 0.269 | 0.017 | 6.959 |
|  | Pars Orbitalis | 7.102 | 0.820 | <0.001 | 6.902 | 0.797 | <0.001 | 1.888 | 0.130 | 1.829 |
|  | Frontal Pole | 7.424 | 0.857 | <0.001 | 7.414 | 0.856 | <0.001 | 0.618 | 0.070 | 10.394 |
| Subcortical | Accumbens | 3.945 | 0.455 | 0.002 | 3.871 | 0.447 | 0.003 | 0.121 | 0.017 | 2.705 |
|  | Pallidum | 5.320 | 0.618 | <0.001 | 5.740 | 0.667 | <0.001 | 1.139 | 0.176 | 1.782 |
|  | Putamen | 4.284 | 0.516 | <0.001 | 3.807 | 0.440 | 0.003 | 0.337 | 0.036 | 2.945 |
|  | Thalamus Proper | 0.800 | 0.093 | 0.847 | 1.695 | 0.196 | 0.540 | 0.116 | 0.008 | 1.807 |
|  | Hippocampus | 2.790 | 0.327 | 0.066 | 3.472 | 0.404 | 0.010 | 0.568 | 0.037 | 3.419 |
|  | Amygdala | 3.216 | 0.371 | 0.022 | 4.510 | 0.521 | <0.001 | 2.185 | 0.262 | 0.289 |
|  | Caudate | 5.925 | 0.684 | <0.001 | 6.532 | 0.759 | <0.001 | 0.568 | 0.088 | 3.419 |

Statistics of cortical gray matter and subcortical areas' LIs at M1 and M2 and comparisons between the two moments. LI=Laterality Index, M1=Moment 1, M2=Moment 2, GM=gray matter, corrected p-value=Bonferroni-Holm corrected p-value for 41 comparisons.
