# Supplementary material for: Asymmetrical subcortical plasticity entails cognitive progression in older individuals
Source: Aging Cell. 2018 Dec 21;18(1):e12857. doi: 10.1111/acel.12857 (PMC6351824; doi:10.1111/acel.12857)
Supplement: Supplementary file 2 [file ACEL-18-e12857-s002.docx]

**Table S2 - Neuropsychological variation (Δcog) influence in laterality categorization for each subcortical area**

|  | | uncorrected | | | | corrected | | | |
| --- | --- | --- | --- | --- | --- | --- | --- | --- | --- |
|  |  | OR | 95% CI | | p-value | OR | 95% CI | | p-value |
|  |  |  | LL | HL |  |  | LL | HL |  |
| SRT-CLTR | Thalamus Proper | 1.003 | 0.952 | 1.057 | 0.909 | 1.014 | 0.958 | 1.073 | 0.634 |
|  | Putamen | 1.015 | 0.967 | 1.065 | 0.552 | 1.006 | 0.954 | 1.061 | 0.826 |
|  | Accumbens | 1.002 | 0.955 | 1.051 | 0.939 | 1.012 | 0.961 | 1.066 | 0.652 |
|  | Amygdala | 0.989 | 0.943 | 1.039 | 0.670 | 0.985 | 0.937 | 1.036 | 0.558 |
|  | Hippocampus | 0.979 | 0.935 | 1.024 | 0.352 | 0.976 | 0.932 | 1.023 | 0.310 |
|  | Pallidum | 0.978 | 0.932 | 1.025 | 0.352 | 0.967 | 0.918 | 1.018 | 0.201 |
|  | Caudate | 1.002 | 0.960 | 1.046 | 0.919 | 1.009 | 0.965 | 1.056 | 0.687 |
| SRT-LTS | Thalamus Proper | 0.989 | 0.939 | 1.040 | 0.659 | 0.993 | 0.941 | 1.047 | 0.786 |
|  | Putamen | 1.013 | 0.959 | 1.071 | 0.636 | 1.000 | 0.939 | 1.063 | 0.988 |
|  | Accumbens | 0.986 | 0.941 | 1.033 | 0.546 | 0.999 | 0.950 | 1.051 | 0.970 |
|  | Amygdala | 0.986 | 0.937 | 1.038 | 0.593 | 0.983 | 0.932 | 1.037 | 0.533 |
|  | Hippocampus | 0.980 | 0.933 | 1.030 | 0.431 | 0.980 | 0.931 | 1.031 | 0.429 |
|  | Pallidum | 0.974 | 0.928 | 1.023 | 0.292 | 0.966 | 0.916 | 1.018 | 0.193 |
|  | Caudate | 0.984 | 0.940 | 1.029 | 0.480 | 0.986 | 0.940 | 1.033 | 0.546 |
| SRT_DR | Thalamus Proper | 1.055 | 0.863 | 1.289 | 0.603 | 1.119 | 0.896 | 1.397 | 0.321 |
|  | Putamen | 1.008 | 0.844 | 1.204 | 0.932 | 1.056 | 0.868 | 1.285 | 0.585 |
|  | Accumbens | 0.927 | 0.755 | 1.138 | 0.468 | 0.968 | 0.777 | 1.206 | 0.771 |
|  | Amygdala | 1.227 | 0.970 | 1.550 | 0.088 | 1.240 | 0.978 | 1.573 | 0.076 |
|  | Hippocampus | 0.845 | 0.683 | 1.045 | 0.121 | 0.840 | 0.675 | 1.044 | 0.115 |
|  | Pallidum | 1.002 | 0.828 | 1.212 | 0.988 | 0.950 | 0.773 | 1.167 | 0.626 |
|  | Caudate | 0.874 | 0.723 | 1.057 | 0.164 | 0.891 | 0.728 | 1.091 | 0.263 |
| Stroop-Golden | Thalamus Proper | 1.045 | 0.997 | 1.096 | 0.068 | 1.048 | 0.998 | 1.100 | 0.060 |
|  | Putamen | 1.018 | 0.969 | 1.070 | 0.475 | 1.027 | 0.977 | 1.081 | 0.294 |
|  | Accumbens | 1.003 | 0.959 | 1.050 | 0.889 | 0.997 | 0.952 | 1.045 | 0.909 |
|  | Amygdala | 1.022 | 0.971 | 1.076 | 0.408 | 1.020 | 0.967 | 1.076 | 0.470 |
|  | Hippocampus | 1.006 | 0.954 | 1.060 | 0.838 | 1.002 | 0.948 | 1.059 | 0.945 |
|  | Pallidum | 1.008 | 0.959 | 1.059 | 0.765 | 1.003 | 0.951 | 1.058 | 0.905 |
|  | Caudate | 0.935 | 0.886 | 0.988 | 0.016 | 0.930 | 0.877 | 0.985 | 0.014 |
| Stroop-Chafetz | Thalamus Proper | 1.051 | 1.002 | 1.102 | 0.040 | 1.053 | 1.003 | 1.106 | 0.037 |
|  | Putamen | 1.033 | 0.980 | 1.089 | 0.232 | 1.039 | 0.985 | 1.095 | 0.162 |
|  | Accumbens | 1.012 | 0.968 | 1.058 | 0.594 | 1.008 | 0.963 | 1.055 | 0.724 |
|  | Amygdala | 1.007 | 0.959 | 1.057 | 0.781 | 1.004 | 0.955 | 1.057 | 0.865 |
|  | Hippocampus | 1.010 | 0.959 | 1.063 | 0.712 | 1.004 | 0.952 | 1.058 | 0.895 |
|  | Pallidum | 0.997 | 0.952 | 1.044 | 0.890 | 0.995 | 0.948 | 1.044 | 0.827 |
|  | Caudate | 0.940 | 0.891 | 0.992 | 0.025 | 0.933 | 0.881 | 0.988 | 0.017 |
| MMSE | Thalamus Proper | 1.210 | 0.927 | 1.579 | 0.160 | 1.278 | 0.963 | 1.697 | 0.089 |
|  | Putamen | 0.889 | 0.689 | 1.147 | 0.365 | 0.884 | 0.676 | 1.156 | 0.368 |
|  | Accumbens | 1.137 | 0.883 | 1.464 | 0.318 | 1.191 | 0.914 | 1.552 | 0.194 |
|  | Amygdala | 1.095 | 0.834 | 1.438 | 0.512 | 1.120 | 0.836 | 1.500 | 0.448 |
|  | Hippocampus | 1.063 | 0.814 | 1.388 | 0.653 | 1.129 | 0.855 | 1.490 | 0.393 |
|  | Pallidum | 1.134 | 0.870 | 1.476 | 0.353 | 1.147 | 0.871 | 1.510 | 0.328 |
|  | Caudate | 1.491 | 1.105 | 2.014 | 0.009 | 1.517 | 1.105 | 2.083 | 0.010 |
| GDS | Thalamus Proper | 1.061 | 0.953 | 1.182 | 0.282 | 1.059 | 0.946 | 1.186 | 0.316 |
|  | Putamen | 0.915 | 0.808 | 1.037 | 0.163 | 0.878 | 0.766 | 1.005 | 0.058 |
|  | Accumbens | 1.029 | 0.924 | 1.145 | 0.604 | 1.032 | 0.925 | 1.150 | 0.572 |
|  | Amygdala | 1.099 | 0.970 | 1.244 | 0.137 | 1.099 | 0.970 | 1.244 | 0.138 |
|  | Hippocampus | 0.997 | 0.896 | 1.111 | 0.963 | 0.960 | 0.855 | 1.078 | 0.489 |
|  | Pallidum | 0.925 | 0.816 | 1.048 | 0.220 | 0.967 | 0.845 | 1.107 | 0.628 |
|  | Caudate | 1.025 | 0.901 | 1.166 | 0.710 | 1.024 | 0.896 | 1.169 | 0.728 |
| DS-D | Thalamus Proper | 0.866 | 0.644 | 1.165 | 0.342 | 0.854 | 0.632 | 1.154 | 0.303 |
|  | Putamen | 0.930 | 0.698 | 1.240 | 0.621 | 0.940 | 0.702 | 1.259 | 0.680 |
|  | Accumbens | 0.932 | 0.696 | 1.248 | 0.634 | 0.892 | 0.660 | 1.206 | 0.457 |
|  | Amygdala | 0.917 | 0.680 | 1.237 | 0.570 | 0.914 | 0.676 | 1.234 | 0.556 |
|  | Hippocampus | 0.924 | 0.705 | 1.212 | 0.568 | 0.926 | 0.700 | 1.224 | 0.588 |
|  | Pallidum | 0.865 | 0.654 | 1.144 | 0.311 | 0.876 | 0.659 | 1.165 | 0.364 |
|  | Caudate | 0.932 | 0.708 | 1.227 | 0.617 | 0.962 | 0.726 | 1.275 | 0.787 |
| DS-B | Thalamus Proper | 0.890 | 0.653 | 1.214 | 0.463 | 0.917 | 0.645 | 1.303 | 0.628 |
|  | Putamen | 0.934 | 0.684 | 1.275 | 0.667 | 0.900 | 0.640 | 1.265 | 0.543 |
|  | Accumbens | 0.863 | 0.639 | 1.167 | 0.340 | 0.857 | 0.613 | 1.198 | 0.367 |
|  | Amygdala | 1.098 | 0.785 | 1.535 | 0.585 | 1.049 | 0.730 | 1.508 | 0.795 |
|  | Hippocampus | 0.969 | 0.711 | 1.321 | 0.842 | 0.899 | 0.636 | 1.270 | 0.545 |
|  | Pallidum | 0.975 | 0.719 | 1.322 | 0.869 | 1.414 | 0.954 | 2.097 | 0.085 |
|  | Caudate | 1.090 | 0.809 | 1.468 | 0.572 | 1.174 | 0.838 | 1.644 | 0.352 |

Results of logistic regression analyses in which the dependent variable is the category for laterality change (left, right and nil) for each subcortical region and the independent variable of interest is cognitive change. Uncorrected analysis (left) was only controlled for gray matter volume change as a proxy for aging, while controlled analysis (right) was corrected for sex, age and cognitive performance group (good or poor performer). SRT=Selective Reminding Test, LTS=long term storage, CLTR=consistent long term retrieval, DR=delayed recall, MMSE=Mini-Mental State Examination, GDS=Geriatric Depression Scale, DS=Digits Span Test, D=direct, B=backward, OR=odd's ratio, CI=confidence interval.
