# Supplementary material for: Asymmetrical subcortical plasticity entails cognitive progression in older individuals
Source: Aging Cell. 2018 Dec 21;18(1):e12857. doi: 10.1111/acel.12857 (PMC6351824; doi:10.1111/acel.12857)
Supplement: Supplementary file 3 [file ACEL-18-e12857-s003.docx]

**Table S3 – Cohort characterization**

| cognitive performance group | good (62.7%) | | poor (37.3%) | |
| --- | --- | --- | --- | --- |
| sex | female (42.6%) | male (57.4%) | female (57.1%) | male (42.9%) |
| age (y) | 66.850±7.842 | 62.815±9.068 | 65.625±6.323 | 63.667±6.050 |
| education (y) | 5.400±3.515 | 8.593±4.643 | 3.188±1.424 | 4.167±2.038 |

The final cohort comprised 75 subjects, here characterized according to cognitive performance group, sex, age and education. Data is shown as mean ± standard deviation. y=years.
